# Poor Glycaemic Control Is Associated with Increased Lipid Peroxidation and Glutathione Peroxidase Activity in Type 2 Diabetes Patients

**DOI:** 10.1155/2019/9471697

**Published:** 2019-08-05

**Authors:** Harshi Prasadini Gunawardena, Renuka Silva, Ramiah Sivakanesan, Pathmasiri Ranasinghe, Prasad Katulanda

**Affiliations:** ^1^Department of Applied Nutrition, Faculty of Livestock, Fisheries & Nutrition, Wayamba University of Sri Lanka, Makandura, Gonawila, 60170, Sri Lanka; ^2^Department of Biochemistry, Faculty of Medicine, University of Peradeniya, Sri Lanka; ^3^Herbal Technology Division, Industrial Training Institute, Sri Lanka; ^4^Department of Clinical Medicine, Faculty of Medicine, University of Colombo, Sri Lanka

## Abstract

Glycaemic control is the main focus of managing diabetes and its complications. Hyperglycaemia induces oxidative stress favouring cellular damage and subsequent diabetic complications. The present study was conducted to compare the plasma total antioxidant capacity (TAC) and individual antioxidant marker antioxidant status of type 2 diabetics (T2D) with good ((+) GC) and poor ((-) GC) glycaemic control with prediabetic (PDM) and normoglycaemic (NG) individuals. T2D (*n* = 147), PDM (*n* = 47), and NGC (*n* = 106) were recruited as subjects. T2D and PDM had lower plasma TAG than NG subjects. T2D and PDM had significantly higher GPx activity and plasma MDA concentrations than NG. PDM showed the highest SOD activity. T2D (-) GC showed significantly elevated GPx activity and higher MDA level and significantly lower SOD activity among all study groups. Lower plasma TAC and higher plasma MDA indicate the presence of oxidative stress in T2D and PDM. Elevated GPx activity in T2D, PDM, and particularly in T2D (-) GC suggests a compensatory response to counteract excess lipid peroxidation in the hyperglycaemic state. Decline in SOD activity advocates the presence of glycation and excess lipid peroxidation in T2D.

## 1. Introduction

Glycaemic control is the central focus of managing diabetes and its complications. Hyperglycaemia promotes oxidative stress through increased glucose autooxidation, protein glycation, and successive degradation of glycated proteins [1]. Excess ROS production and subsequent decline in cellular antioxidant defense facilitate the rise in systemic insulin resistance and lipid peroxidation leading to cellular damage and cytoprotective enzyme inactivation [2, 3].

Consequently, systemic oxidative stress and accompanied defects in antioxidant defense mechanisms promote the predisposition of diabetic complications, both micro and macro vascular [4]. Earlier tight glucose control has shown to improve cardiovascular morbidity in the United Kingdom Prospective Diabetes Study (UKPDS) [5]. Further, a recent follow-up to the same study was able to verify ability of long-term glycaemic control in type 2 diabetes in preventing the cardiovascular diseases [6]. However, the role of glycaemic control in improving the diabetes complications via oxidative stress pathways is not adequately inquired.

Previous findings on glycaemic control and oxidative stress in patients with type 2 diabetes (T2D) are ambiguous. There were depletions [7–15], improvements [16–20], and no change [21] in the total antioxidant status, and antioxidant enzyme activities in diabetics with poor glycaemic control have been disclosed. Therefore, diabetes patients with good and poor glycaemic control were used to address the unsolved interpretations of glycaemic control on plasma antioxidant capacity and antioxidant markers.

## 2. Materials and Methods

### 2.1. Subjects

One hundred and forty-seven (*n* = 147) type 2 diabetics (T2D), forty-seven (*n* = 47) prediabetics (PDM), and a hundred and six (*n* = 106) normoglycaemic (NG) apparently healthy individuals were recruited as subjects.

T2D patients were recruited from the diabetes clinical registries of selected Government Hospitals of North Western Province, Sri Lanka. Screening sessions were conducted in community centers and working places situated closer to the Wayamba University of Sri Lanka to identify PDM and NG individuals. Screened individuals were invited to participate in the study. Individuals who were already diagnosed by a clinician or identified by the researchers in screening sessions having impaired fasting glycaemia (6.1–6.9 mmol/L) [22] more than two consecutive occasions verified with glycated haemoglobin (HbA1c) measurement greater than 6% were considered as the PDM. Individuals with a fasting plasma glucose level below 6.1 mmol/L were considered as normoglycaemic [22].

T2D, PDM, and NG volunteers with known history of heart disease, liver disease, kidney diseases, or endocrine diseases other than diabetes and those who were ill for more than seven days during the last three months were excluded. Smokers, regular users of alcohol (more than 2 times per week), those who followed a weight loss regimen, users of antioxidant supplements (vitamins and minerals), and pregnant and lactating mothers were also excluded. None of the T2D patients selected were on insulin for glycaemic control during the study period.

#### 2.1.1. Ethics

The study was approved by the ethics review committee of Sri Lanka Medical Association (ERC/07/007). The study protocol was in accordance with the principles described in the Declaration of Helsinki. Written informed consent was obtained from each participant after providing the detailed description of the study. Prior approval from the respective hospital authorities was taken for recruiting diabetics.

### 2.2. Data Collection

Details of general life style, disease, and medical history of the subjects were collected using a pretested questionnaire. Weight and height of the subjects were measured using standard methods in light indoor clothing. The waist circumference of the subjects was measured around the point of the umbilicus using a nonstretchable plastic tape. The hip circumference was measured at the widest point of the body.

#### 2.2.1. Systolic and Diastolic Blood Pressures

Seated systolic (SBP) and diastolic (DBP) blood pressures were measured in the nondominant arm using a digital sphygmomanometer (Omron Ltd., Singapore) after resting for 10 minutes prior to the measurement. Average of the last two readings was considered. Subjects were refrained from speaking and moving during the measurement.

### 2.3. Blood Collection and Biochemical Analysis

#### 2.3.1. Blood Collection

A single 10 mL of fasting venous blood sample was drawn by a phlebotomist from each participant following 12 hours of fasting. The blood sample was then transferred to blood collection tubes containing lithium heparin for glutathione peroxidase (GPx), superoxide dismutase (SOD), Trolox equivalent antioxidant capacity (TEAC) assay, oxygen radical absorption capacity (ORAC) assay, and malondialdehyde (MDA) analysis; EDTA for ferric reducing ability of plasma (FRAP) assay, HbA1c, fasting plasma glucose, and fasting lipids; and serum tubes for fasting serum insulin analysis.

Aliquots of whole blood were stored at -80°C for future analysis of erythrocyte GPx and SOD. For HbA1c determination, aliquot of whole blood was stored at -20°C. Heparinized plasma was separated for further analysis of TEAC, ORAC, and MDA using a centrifuge set at 4000 rpm for 10 minutes and stored at -80°C. Separated EDTA plasma was stored at -80°C for FRAP analysis. For the fasting plasma glucose, lipid profile, and insulin analyses, aliquots of plasma and serum were stored at -20°C.

#### 2.3.2. Biochemical Analysis

*(1) Plasma Total Antioxidant Capacity (TAC)*. *Plasma FRAP assay*. The plasma FRAP assay was performed according to the method described by Benzie and Strain [23]. Briefly, 3 mL of a working FRAP reagent was mixed with 100 *μ*L of plasma followed by mixing and incubating for 6 minutes at 37°C. After the 6 minutes of incubation, absorption was read by a spectrophotometer (Labo Med Inc., UK) set at 593 nm. Plasma FRAP values were expressed as *μ*mol of FeSO_4_ equivalents per litre.

*Plasma TEAC assay*. Plasma TEAC was measured using the method described by Re et al. [24]. ABTS radical cation was prepared by mixing equal volumes of 7 mM ABTS (2,2′-azino-bis(3-ethylbenzo-thiazoline-6-sulfonic acid)) and 2.45 mM potassium persulfate solutions and keeping it for 16-24 hours in the dark. ABTS radical cation was diluted with PBS (pH 7.4) for an absorbance of 0.7 (±0.02) and equilibrated at 30°C. Then, 100 *μ*L of diluted plasma was added to the ABTS cation solution and incubated for 6 minutes at 30°C. Absorbance readings were taken at one-minute intervals after initial mixing up to 6 min using a spectrophotometer set at 734 nm. Plasma TEAC values were expressed in Trolox equivalents per litre.

*Plasma ORAC assay*. Plasma ORAC was measured as the method described by Cao et al. [25]. Diluted heparinized plasma was mixed with 100 *μ*L of fluorescein in black plates followed by preheating in the microplate reader set at 37°C for 10 minutes. After the addition of 50 *μ*L of AAPH solution and mixing, reading was taken at one-minute intervals using a microplate reader set at excitation and emission wavelengths of 494 and 535 nm, respectively. The net area under the curve for each standard and sample was derived, and ORAC values were expressed as Trolox units per litre.

*Erythrocyte GPx and SOD activities*. Activities of erythrocyte glutathione peroxidase (GPx) (RANSEL RS 505, Randox Laboratories, UK) and superoxide dismutase (SOD) (RANSOD SD 125, Randox Laboratories, UK) were measured using the enzymatic reagent kits, following the manufacturer's instructions. The activities of both enzymes were normalized by the concentration of haemoglobin (Hb) and expressed as units per gram of Hb per min.

*Plasma MDA level*. Plasma lipid peroxidation marker, malondialdehyde (MDA), concentration was measured as a marker of lipid peroxidation using thiobarbituric acid reactive substances (TBARS) [26]. Briefly, 200 *μ*L of heparinized plasma was first mixed with Butylated Hydroxytoluene (BHT), and plasma proteins were precipitated by adding trichloroacetic acid (TCA). Then, the thiobarbituric acid (TBA) was added to the mixture and incubated on a water bath set at 95°C for 45 minutes. The resulting chromogen was extracted using n-butanol and saturated NaCl solution. The organic mixture was separated by centrifugation. Intensity of the upper organic layer was measured spectrophotometrically (model: UV/Visible Auto PC Scanning spectrophotometer; Labo Med Inc., Germany) at 535 nm and 572 nm against the blank. The MDA level of the samples was determined using the linear standard curve developed by 0.5 to 32 *μ*M of 1,1,3,3-tetraethoxypropane.

*Glycaemic control*. Fasting plasma glucose (FPG) and HbA1c levels of the subjects were measured using enzymatic reagent kits (Stanbio, USA). Fasting insulin was analyzed by an Enzyme-Linked Immunosorbent Assay (ELISA) kit (DRG, USA). Insulin resistance was derived using the Homeostatic Model Assessment of Insulin Resistance (HOMA-IR) by multiplying fasting plasma glucose (mmol/L) and fasting insulin level (pmol/L) and dividing it by 135.

*Fasting plasma lipids*. Fasting total cholesterol and triacylglycerol levels were measured using the enzymatic reagent kits (Stanbio, USA). HDL cholesterol was determined using an enzymatic reagent kit after selective precipitation of VLDL and LDL [27]. The LDL cholesterol level was estimated using the Friedewald formula [28].

### 2.4. Data Analysis

T2D and PDM were further clustered into two groups based on the presence or absence of good glycaemic control (HbA1c < 7%; (+) GGC) and poor glycaemic control (HbA1c > 7%; (-) GGC).

BMI > 25 kg m^−2^ was used to define obesity (WHO Definition for BMI of Asians, 2004). The waist circumference (>85 cm for women and >90 for males) and waist to hip ratio (WHR > 0.85 for females and 0.9 for males) (WHO, 2008) were used to define central obesity. Metabolic syndrome was defined according to the International Diabetes Federation Definition for metabolic syndrome [29].

### 2.5. Statistical Analysis

Data were expressed in absolute numbers and percentages. Data were checked for normality using the Kolmogorov-Smirnov test. If the data of a variable were not normally distributed, they were transformed to natural logarithms and checked for normality, and a parametric test was used. One-way ANOVA was used in order to observe the differences in antioxidant markers and anthropometric and biochemical variables between groups. Effects of the study group and glycaemic control on total antioxidant status and individual antioxidant markers were assessed using factorial ANOVA with multiple comparisons. Post hoc tests (Tukey's *b* with Bonferroni correction) were performed to compare the subgroups with and without good glycaemic control for their total antioxidant status and individual antioxidant markers. Data were analyzed using the SPSS (Statistical Package for Social Sciences), version 16.

## 3. Results

General characteristics of the study subjects are shown in Table 1. Average clinical duration of diabetes among T2D was 5.0 ± 4.6 years. Nearly 99% of T2D were on oral hypoglycaemic agents (OHA) for glycaemic control. Only 1-2% reported that they were on dietary and physical activity modifications for glycaemic control. None of the T2D was on insulin for glycaemic control. Biguanide (metformin) was the main type (93%) of OHA used. In addition, 46% of diabetics used sulfonylureas (tolbutamide, glibenclamide, and Diaonil) along with biguanides. None of the PDM was on OHA for glycaemic control. However, only 61% of T2D and 66% of PDM were on proper glycaemic control (HbA1c < 7.0). Thirty-one percent (31%) of T2D were hypertensive and on antihypertensive drugs. The presence of dyslipidemia among T2D was 20%, and all were on lipid-lowering drugs (statins). Twenty-three percent (23%) of PDM and 12% of NG had dyslipidemia, and they were not on drug therapy.

T2D had a significantly (*P* < 0.05) higher waist to hip ratio (WHR), SBP, FPG, and HOMA-IR than the PDM and NG groups (Table 1). Both T2D and PDM had significantly higher (*P* < 0.05) HbA1c and plasma TAG compared with the NG group (Table 1).

### 3.1. Antioxidant Status

Table 2 shows the antioxidant status of T2D, PDM, and NG groups. T2D had significantly (*P* < 0.05) lower plasma FRAP, TEAC, and ORAC compared to NG. T2D and PDM had similar plasma TEAC and ORAC. However, the T2D group had significantly (*P* < 0.05) higher erythrocyte GPx activity. Significantly (*P* < 0.05) higher SOD activity was shown by the PDM than T2D and NG groups.

Table 3 shows the results of correlation analysis between antioxidant markers and glycaemic control parameters of T2D and PDM groups. Plasma ORAC was negatively correlated with FPG, HbA1c of T2D, and HOMA-IR of PDM. GPx activity had a positive correlation with HbA1c of T2D. SOD activity showed negative relationship with FPG, HbA1c in T2D, and FPG in PDM (Table 3).

Effects of glycaemic control on plasma TAC and other antioxidant markers are given in Table 4. PDM subgroup with good glycaemic control had significantly (*P* < 0.05) lower TEAC compared to all the other subgroups. PDM subgroup without good glycaemic control and NG group had significantly (*P* < 0.05) higher plasma TEAC than T2D and PDM subgroups with good glycaemic control. A significantly (*P* < 0.05) higher ORAC was observed in NG compared to T2D subgroups with and without good glycaemic control (Table 4).

**Figure 1:**
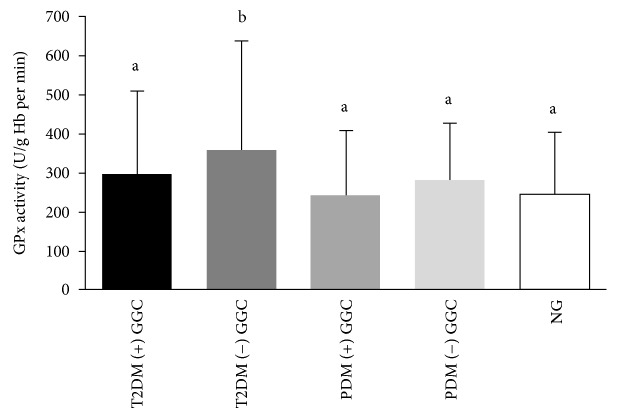
Erythrocyte GPx activity among subgroups with good glycaemic control ((+) GC) and without good glycaemic control ((-) GC) of T2D, PDM, and NG study groups. Means with the different superscripts are significantly different at *P* < 0.05 (Tukey's post hoc test).

**Figure 2:**
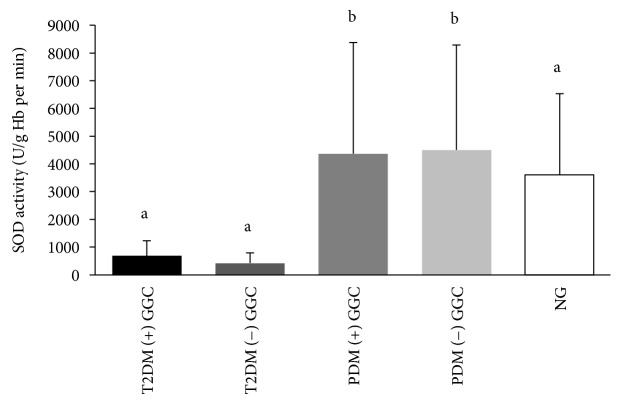
Erythrocyte SOD activity among subgroups with good glycaemic control ((+) GC) and without good glycaemic control ((-) GC) of T2D, PDM, and NG study groups. Means with the different superscripts are significantly different at *P* < 0.05 (Tukey's post hoc test).

**Figure 3:**
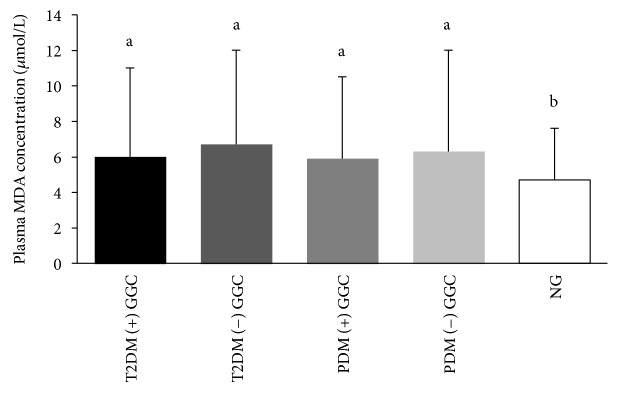
Plasma MDA concentration among subgroups with good glycaemic control ((+) GC) and without good glycaemic control ((-) GC) of T2DM, PDM, and NG study groups. Means with the different superscripts are significantly different at *P* < 0.05 (Tukey's post hoc test).

Effects of glycaemic control on erythrocyte GPx and SOD activities and plasma MDA concentration are illustrated in Figures 1–3, respectively. T2D subgroup without good glycaemic control had significantly higher GPx activity compared with the PDM subgroups with and without good glycaemic control and the NG group (Figure 1).

PDM subgroups had the highest SOD activity, and they were significantly higher than T2D and NG. The lowest SOD activity was observed in T2D subgroups, and it was significantly lower than other subgroups of PDM and NG (Figure 2).

There was no significant effect of glycaemic control on plasma MDA concentration. However, the NG group had the significantly lowest level of plasma MDA compared to T2D and PDM subgroups with and without good glycaemic control (Figure 3).

## 4. Discussion

Both T2D and PDM groups had lower plasma TAC compared with the NG subjects. Hyperglycaemia induces the excess ROS generation thus depleting the plasma TAC in T2D and PDM [30–33]. T2D and PDM subgroups with poor glycaemic control had depleted plasma TAC compared to the NG similar to previous studies [9, 31].

T2D and PDM subjects exhibited higher levels of the plasma lipid peroxidation marker, MDA. The rise in plasma MDA among T2D has been previously reported similar to our findings [34, 35]. Hyperglycaemia promotes ROS-mediated lipid peroxidation via nonenzymatic and autoglycation pathways [1, 7]. Hyperlipidemia experienced by the T2D and PDM could be one of the reasons for increased production of lipid peroxides [7]. Although we could not obtain significantly different MDA levels in T2D with good and poor glycaemic control, previous authors have demonstrated raised MDA levels among T2D with poor glycaemic control [35, 36]. It also highlights the presence of lipid peroxidation and systemic inflammation that contributes to the metabolic imbalance even in well-controlled T2D as suggested by previous authors [37, 38].

Hence, depleted plasma TAC and increased MDA represent the presence of oxidative stress in T2D and PDM of the present study. MDA levels of T2D and PDM were positively correlated with fasting plasma glucose and HOMA-IR and therefore show the association of hyperglycaemia with oxidative stress. It further verifies that persistent hyperglycaemia promotes ROS production, leading to lipid peroxidation and depletion of endogenous antioxidants demonstrating the increased oxidative stress in T2D [39]. Differences in sample sizes of T2D and PDM and use of oral hypoglycaemic agents only by the T2D might have contributed to the identical HbA1c levels in T2D and PDM of the present study.

Erythrocyte GPx activity has elevated in T2D and PDM and particularly the T2D and PDM with poor glycaemic control. GPx responses in T2D were controversial [15, 40–42]. Our findings on GPx activity are in agreement with previous authors [40, 41]. GPx is thought to represent the initial protective response required for adjusting hydrogen peroxide concentration in normal physiological conditions and after oxidative insult [43]. In the present study, the rise of erythrocyte GPx activity in T2D and PDM was parallel to that of MDA. Therefore, we can hypothesize that the excess hydrogen peroxide generation resulted from lipid peroxidation in T2D and PDM, which may have stimulated the GPx activity.

Further, T2D and PDM with poor glycaemic control showed the higher GPx activity parallel to the MDA levels. T2D with poor glycaemic control had the highest GPx activity in the present study. As the primary function of GPx is to achieve the hydrogen peroxide balance, once the hydrogen peroxide production is exaggerated via the lipid peroxidation pathway, GPx activity would have been raised. It suggests an adaptive response against the excess lipid peroxidation and accumulation of hydrogen peroxide in a dysglycaemic state [44]. Therefore, the T2D with poor glycaemic control tend to exhibit the higher GPx activity among all groups. Positive correlations existed between HbA1c and GPx activity; FPG and MDA of T2D further verify the above findings.

In contrast to the GPx activity, SOD activity was intensely reduced in T2D irrespective of their glycaemic control. PDM had the highest erythrocyte SOD activity in the present study. Blunting of the SOD activity due to the poor glycaemic control has already been studied and proven by previous studies [15, 45, 46]. Our findings are also in agreement with previous findings [15, 45, 46] as shown by negative relationship between the HbA1c and SOD activity in T2D. Low activity of the SOD can be partly explained by the enzymatic glycation of SOD due to the higher circulating levels of glucose [15]. Nearly 50% of SOD in diabetics are glycated and made the SOD inactive [47]. In addition, excess hydrogen peroxide produced in lipid peroxidation and autooxidation of glucose has contributed to the lower SOD activity in T2D [48].

However, the upsurge of SOD among PDM suggests an endogenous adaptive response on metabolic alterations underneath the prediabetic stage [49]. Less exposure to the hyperglycaemic spikes by the PDM than T2D might have not suppressed the SOD activity. Increased production of superoxide radical and an accompanied rise of hydrogen peroxide may be the reason for exaggerated SOD activity in PDM. A proposed mechanism is further supported by the increased GPx activity in PDM.

Hence, the upsurge of erythrocyte GPx activity in parallel to the lipid peroxidation in T2D and PDM and particularly in T2D with poor glycaemic control drives the adaptive response for counteracting the excess hydrogen peroxide and other free radicals generated. Glycation and excess accumulation of hydrogen peroxide have made the part of SOD inactive in T2D. The rise in SOD among PDM suggests the potential metabolic adaptation. Increased GPx activity in T2D and PDM and SOD activity in PDM suggest the concept of reductive stress as a temporary defense against the oxidative stress in hyperglycaemia.

Reductive stress followed by oxidative stress may serve as one of the major mechanisms of glucotoxicity in a persistent hyperglycaemic state [50]. Hence, the oxidative stress is believed to be originated from NADH-imposed reductive stress thus would remain temporary [51]. Attenuated hyperglycaemia-induced reductive stress may provide therapeutic approaches for preventing diabetes and diabetic complications [50].

Almost all of the T2D of the present study were on oral hypoglycaemic agents for glycaemic control. Metformin and gliazide drugs of the sulphonylurea group were shown to improve the antioxidant status of T2D [52, 53]. Further, the use of antihypertensive drugs [54, 55] and statins can [56, 57] enhance the antioxidant status of T2D. Therefore, the use of oral hypoglycaemic agents, particularly the metformin, antihypertensive drugs, and statins, might have contributed to the pronounced antioxidant activities shown by T2D. However, the life style pattern (dietary and physical activity patterns) of subjects may have exerted beneficial effects on antioxidant status. Therefore, it would be further useful to assess the contribution of the life style pattern on antioxidant status.

## 5. Conclusions

T2D and PDM had depleted plasma TAC compared to the NG. It suggests that the hyperglycaemia induced ROS generation and overuse of endogenous antioxidants available in plasma. Poor glycaemic control has further shown to deteriorate the plasma TAC of T2D and PDM. Lower plasma TAC and higher plasma MDA levels further confirm the presence of oxidative stress in T2D and PDM. Increased erythrocyte GPx activity found in T2D and PDM and further rise of GPx in T2D and PDM with poor glycaemic control suggest a compensatory adaptive response for poor glycaemic control and accompanied excess lipid peroxidation. Hence, the parallel rise of the lipid peroxidation marker and GPx activity is seen. Decline in SOD activity advocates the excess hydrogen peroxide generation due to glucose autoxidation, lipid peroxidation, and glycation present in hyperglycaemic status.

## Figures and Tables

**Table 1:** Anthropometric, clinical, and biochemical characteristics of the study subjects (*n* = 300).

Variable	T2D (*n* = 147)	PDM (*n* = 47)	NG (*n* = 106)	*P*
Age (years), mean ± SD	47.6 ± 8.3	45.7 ± 8.8	44.2 ± 8.2	*0.125* ^1^
Sex, M/F (%)	42/58	48/52	42/58	*0.236* ^2^
BMI (kg m^−2^), mean ± SD	24.7 ± 4.3	24.9 ± 4.1	25.2 ± 3.2	*0.156* ^1^
WC (cm), mean ± SD	87.7 ± 8.8	87.0 ± 9.1	85.0 ± 8.0	*0.143* ^1^
WHR, mean ± SD	0.96^a^ ± 0.07	0.93^b^ ± 0.06	0.92^b^ ± 0.06	*0.033* ^1^
Diabetes duration (years), mean ± SD	5.0 ± 4.6	—	—	
SBP (mmHg), mean ± SD	127^a^ ± 19	121^b^ ± 17	121^b^ ± 16	*0.043* ^1^
DBP (mmHg), mean ± SD	77 ± 13	76 ± 13	76 ± 11	*0.237* ^1^
FPG (mmol/L), mean ± SD	7.9^a^ ± 2.80	5.9^a^ ± 1.20	5.1^a^ ± 0.56	*0.031* ^1^
HbA1c (%), mean ± SD	6.70^a^ ± 1.50	6.70^a^ ± 1.00	5.10^b^ ± 0.70	*0.037* ^1^
TC (mmol/L), mean ± SD	4.6^a^ ± 1.00	4.6^a^ ± 0.70	4.50^a^ ± 1.00	*0.129* ^1^
HDL-C (mmol/L), mean ± SD	0.94^a^ ± 0.27	0.90^a^ ± 0.21	0.92^a^ ± 0.23	*0.078* ^1^
LDL-C (mmol/L), mean ± SD	3.30^a^ ± 1.00	3.40^a^ ± 0.70	3.30^a^ ± 1.00	*0.123* ^1^
TAG (mmol/L), mean ± SD	1.60^a^ ± 0.78	1.60^a^ ± 0.85	1.30^b^ ± 0.62	*0.033* ^1^
HOMA-IR, mean ± SD	3.60^a^ ± 4.00	2.70^b^ ± 2.80	2.0^b^ ± 2.50	*0.029* ^1^
Family history of T2DM (%)	51^a^	41^a^	32^b^	*0.041* ^2^
Family history of HTN (%)	39^a^	37^a^	21^b^	*0.043* ^2^
Family history of CVD (%)	11	09	13	*0.214* ^2^
Metabolic syndrome (%)	78^a^	52^b^	47^b^	*0.037* ^2^

^1^One-way ANOVA; ^2^Pearson's *χ*^2^ test. Values expressed in absolute numbers and percentages. Means with the different superscripts in a given row are significantly different at *P* < 0.05 (Tukey's post hoc test). BMI: body mass index; DBP: diastolic blood pressure; FPG: fasting plasma glucose; HbA1c: glycated haemoglobin concentration; HDL-C: high-density lipoprotein cholesterol; HOMA-IR: homeostatic model assessment for insulin resistance; LDL-C: low-density lipoprotein cholesterol; NG: normoglycaemic; PDM: prediabetic; SBP: systolic blood pressure; TC: total cholesterol; WC: waist circumference; WHR: waist to hip ratio; T2DM: type 2 diabetes mellitus.

**Table 2:** Plasma total antioxidant capacity and antioxidant markers of the study groups (*n* = 300).

Variable	T2D (*n* = 147)	PDM (*n* = 47)	NG (*n* = 106)	*P* ^1^
Plasma TAC				
FRAP (*μ*mol/L)	787^a^ ± 180	857^b^ ± 211	847^b^ ± 215	*0.033*
TEAC (*μ*mol/L)	1216^a^ ± 484	1180^a^ ± 388	1436^b^ ± 592	*0.027*
ORAC (*μ*mol/L)	4496^a^ ± 1523	4555^a^ ± 2042	5072^b^ ± 1904	*0.019*
GPx activity (U/g Hb/min)	326^a^ ± 249	279^b^ ± 203	244^b^ ± 159	*0.023*
SOD activity (U/g Hb/min)	797^a^ ± 560	4418^b^ ± 3888	3611^c^ ± 2918	*0.011*
Albumin (g/dL)	5.8 ± 1.0	5.5 ± 1.2	5.7 ± 1.9	*0.072*
Bilirubin (mg/dL)	0.9 ± 0.5	0.9 ± 0.2	0.9 ± 0.8	*0.134*
Total protein (g/dL)	7.1^a^ ± 1.5	7.9^a^ ± 1.8	6.5^b^ ± 1.2	*0.047*
Uric acid (*μ*mol/L)	283^a^ ± 72	398^b^ ± 179	387^b^ ± 146	*0.031*
MDA (*μ*mol/L)	6.4^a^ ± 5.1	6.0^a^ ± 5.7	4.7^b^ ± 2.9	*0.036*

All values are mean ± SD. ^1^One-way ANOVA. Means with the different superscripts in a given row are significantly different at *P* < 0.05 (Tukey's post hoc test). NS: not significant; FRAP: ferric reducing antioxidant power assay; GPx: glutathione peroxidase; MDA: malondialdehyde; NG: normoglycaemic subjects; ORAC: oxygen radical absorption capacity; PDM: prediabetics; TEAC: Trolox equivalent antioxidant capacity assay; TAC: total antioxidant capacity; T2DM: type 2 diabetes mellitus.

**(a):** 

Parameter	T2D group (*n* = 147)					
	Ln FRAP	TEAC	ORAC	GPx	SOD	MDA
FPG	-0.08	0.08	-0.20^∗^	0.02	-0.20^∗^	0.20^∗^
Ln HbA1c	0.10	0.02	-0.20^∗^	0.20^∗^	-0.20^∗^	0.02
HOMA-IR	0.15	-0.14	0.13	-0.08	-0.06	-0.13

**(b):** 

Parameter	PDM group (*n* = 47)					
	Ln FRAP	Ln TEAC	Ln ORAC	GPx	SOD	MDA
FPG	-0.07	-0.28^∗^	0.11	0.14	0.15^∗^	0.14
Ln HbA1c	-0.30^∗^	0.30^∗^	-0.10	0.06	0.12	0.03
HOMA-IR	0.20^∗^	-0.18	-0.30^∗^	0.11	0.04	0.24^∗^

^∗^Correlations significant at *P* < 0.05. FPG: fasting plasma glucose; HbA1c: glycated haemoglobin level; HOMA-IR: insulin resistance; PDM: prediabetics; T2D: type 2 diabetics.

**Table 4:** Plasma total antioxidant capacity and antioxidant markers of subgroups with and without good glycaemic control (*n* = 300).

Variable	T2D				PDM				NG (*n* = 106)		*P* ^1^
	(+) GC (*n* = 97)		(-) GC (*n* = 50)		(+) GC (*n* = 31)		(-) GC (*n* = 16)				
	Mean	SD	Mean	SD	Mean	SD	Mean	SD	Mean	SD	
*Plasma TAC*											
FRAP (*μ*mol/L)	773	181	812	177	883	205	813	220	847	215	*0.317*
TEAC (*μ*mol/L)	1200^a^	440	1240^ab^	650	1120^c^	450	1320^b^	446	1400^b^	490	*0.007*
ORAC (*μ*mol/L)	4509^a^	15182	4146^a^	1299	4594^ab^	2051	4476^ab^	2093	5156^b^	1844	*0.004*
*Antioxidant markers*											
Uric acid (*μ*mol/L)	288^a^	75	278^a^	70	384^b^	135	429^b^	240	387^b^	146	*0.005*
Albumin (g/L)	5.8	1.0	5.8	1.0	5.5	1.2	5.4	1.0	5.7	1.9	*0.254*
Bilirubin (g/L)	0.90	0.24	0.80	0.22	0.81	0.20	0.90	0.80	0.90	0.80	*0.323*
Total protein (g/L)	7.1^a^	1.4	7.2^a^	1.6	8.0^b^	1.7	7.6^b^	1.8	6.5^c^	1.2	*0.007*

^1^One-way ANOVA. Means with the different superscripts in a given row are significantly different (Tukey's post hoc *t*-test). (+) GC: with good glycaemic control; (-) GC: without good glycaemic control (poor glycaemic control); FRAP: ferric reducing ability of plasma; NG: normoglycaemic; ORAC: oxygen radical absorptive capacity; PDM: prediabetics; TEAC: Trolox equivalent antioxidant capacity; T2D: type 2 diabetics.

## Data Availability

The biochemical and anthropometric data used to support the findings of this study are available from the corresponding author upon request.
